# Saponin 1 Induces Apoptosis and Suppresses NF-κB-Mediated Survival Signaling in Glioblastoma Multiforme (GBM)

**DOI:** 10.1371/journal.pone.0081258

**Published:** 2013-11-21

**Authors:** Juan Li, Haifeng Tang, Yun Zhang, Chi Tang, Bo Li, Yuangang Wang, Zhenhui Gao, Peng Luo, Anan Yin, Xiaoyang Wang, Guang Cheng, Zhou Fei

**Affiliations:** 1 Department of Neurosurgery, Xijing Institute of Clinical Neuroscience, Fourth Military Medical University, Xi’an, China; 2 Department of Pharma of Xijing Hospital, Fourth Military Medical University, Xi’an, China; 3 Department of Immunology, Fourth Military Medical University, Xi’an, China; 4 Faculty of Biomedical Engineering, Fourth Military Medical University, Xi’an, China; University of Nebraska Medical Center, United States of America

## Abstract

Saponin 1 is a triterpeniod saponin extracted from *Anemone taipaiensis*, a traditional Chinese medicine against rheumatism and phlebitis. It has also been shown to exhibit significant anti-tumor activity against human leukemia (HL-60 cells) and human hepatocellular carcinoma (Hep-G2 cells). Herein we investigated the effect of saponin 1 in human glioblastoma multiforme (GBM) U251MG and U87MG cells. Saponin 1 induced significant growth inhibition in both glioblastoma cell lines, with a 50% inhibitory concentration at 24 h of 7.4 µg/ml in U251MG cells and 8.6 µg/ml in U87MG cells, respectively. Nuclear fluorescent staining and electron microscopy showed that saponin 1 caused characteristic apoptotic morphological changes in the GBM cell lines. Saponin 1-induced apoptosis was also verified by DNA ladder electrophoresis and flow cytometry. Additionally, immunocytochemistry and western blotting analyses revealed a time-dependent decrease in the expression and nuclear location of NF-κB following saponin 1 treatment. Western blotting data indicated a significant decreased expression of inhibitors of apoptosis (IAP) family members,(e.g., survivin and XIAP) by saponin 1. Moreover, saponin 1 caused a decrease in the Bcl-2/Bax ratio and initiated apoptosis by activating caspase-9 and caspase-3 in the GBM cell lines. These findings indicate that saponin 1 inhibits cell growth of GBM cells at least partially by inducing apoptosis and inhibiting survival signaling mediated by NF-κB. In addition, *in vivo* study also demonstrated an obvious inhibition of saponin 1 treatment on the tumor growth of U251MG and U87MG cells-produced xenograft tumors in nude mice. Given the minimal toxicities of saponin 1 in non-neoplastic astrocytes, our results suggest that saponin 1 exhibits significant *in vitro* and *in vivo* anti-tumor efficacy and merits further investigation as a potential therapeutic agent for GBM.

## Introduction

Glioblastoma multiforme (GBM, WHO IV) represents approximately 30% of all types of primary brain tumors and remains the leading cause of cancer related death induced by malignant intracranial diseases [1]. Ongoing studies have developed new therapeutic approaches, such as the NovoTTF-100A System [2] and chemotherapeutic agents, such as temozolomide [3], for glioblastoma treatment. However, the clinical benefits are still insufficient because genetic heterogeneity is observed in individual patients and the effectiveness of current chemotherapeutic agents is based on single molecular targets, which can be gradually overcomed as a result of compensation from alternative pro-survival signaling pathways [4]. Therefore, there is an unmet need to develop novel chemotherapeutic agents that target multiple molecular pathways to inhibit pro-survival signals and induce apoptosis in the treatment of glioblastoma multiforme (GBM).

Recently, many active compounds have shown promising chemopreventive and radiosensitizing properties. For example, resveratrol is a natural phenol extracted from red grape skin that shows significant anti-cancer potency in various types of cancer, including breast [5], ovarian [6], and brain cancer [7]. In our previous studies, we isolated a set of saponins from *Ardisia pusilla* A.DC, such as ardipusilloside-I [8] and -III [9], which inhibited cell proliferation and induced apoptosis in pulmonary carcinoma cells and glioblastoma cells. In addition, our previous data suggested that the molecular biochemical mechanisms underlying the anti-cancer activities of these compounds were complex and worked in a network fashion. It has been shown that the inactivation of many essential enzymes in the pro-survival signaling pathways, including the Phosphoinositide 3-kinase (PI3K)/Protein Kinase B (Akt)/mammalian target of rapamycin (mTOR) signaling pathway [10] and the NF-κB signaling pathway, and the down-regulation of some key apoptotic mediators in the IAP family and Bcl-2 family contribute principally to the inhibition of cancer cell proliferation and the induction of apoptosis by these active compounds.

The rhizome of *Anemone taipaiensis* is traditionally used to treat rheumatism and phlebitis in China. Chemical studies on this plant have led to the isolation of eight triterpenoid saponins. Among them, saponin 1, which has a molecular formula of C_46_H_74_O_15_ and molecular weight of 866, exhibited significant cytotoxicity against human leukemia HL-60 cells and human hepatocellular carcinoma Hep-G2 cells [11]. In this study, we investigated the ability of saponin 1 to induce apoptosis in human glioblastoma U251MG and U87MG cells. In addition, we investigated the molecular mechanisms involved in cancer cell apoptosis.

## Materials and Methods

### Plant material and extraction, isolation and characterization of saponin 1

**Figure 1:**
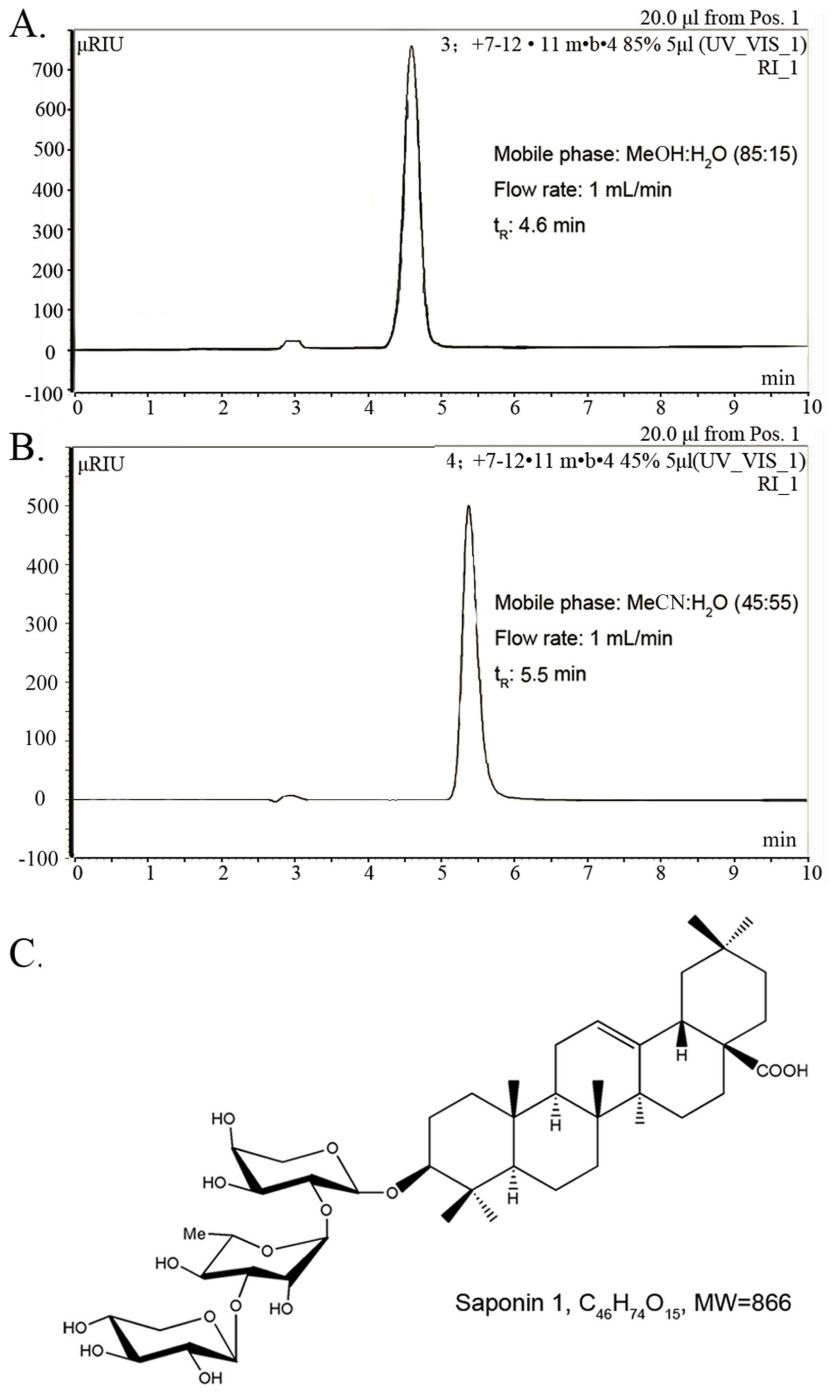
Chemical structure and HPLC analysis of saponin 1. A and B: HPLC with different solvent conditions was carried out to establish the purity of saponin 1 on a Dionex P680 liquid chromatograph equipped with a UV170 UV/Vis detector using a YMC-Pack R&D ODS-A column (20×250 mm, YMC Co., Ltd). C: Chemical structure of saponin 1.

The plant material was collected on Taibai Mountain, Shaanxi Province, China, in September 2009, and was identified as Anemone taipaiensis by Prof. Ji-Tao Wang (Department of Pharmacognosy, School of Pharmacy, Shaanxi University of Chinese Medicine). A voucher specimen (NO.090918) was deposited in the Herbarium of Shaanxi University of Chinese Medicine. The air-dried rhizomes of *A. taipaiensis* (5 kg) (Table S1) were powdered and extracted with 70% EtOH (5 L × 3, 2 h/time) under reflux. The extract was concentrated under vacuum to give a residue (650 g) which was suspended in H_2_O (8 L) and partitioned successively with petroleum ether (8 L × 2) and *n*-BuOH (8 L × 3). The *n*-BuOH extract (110 g) was subjected to column chromatography on silica gel (2200 g, 15 × 120 cm) and eluted with a CHCl_3_-MeOH-H_2_O gradient (10: 1: 0.05, 9: 1: 0.1, 8: 2: 0.2, 7: 3: 0.5, 6: 4: 0.8) to give 9 fractions (Fr.1-Fr.9). Fr.6 (4.5 g) was chromatographed on silica gel (150 g, 4 × 70 cm) with a chloroform- *n*-BuOH gradient (6: 1, 5: 1, 4: 1, 3: 1) to yield saponin 1(120 mg). The purity of saponin 1 was analyzed by high performance liquid chromatography (HPLC) as more than 98% using MeOH:H_2_O(85:15) and MeCN:H_2_O(45:55) as mobile phase, respectively (Figure 1A and B). On the basis of its spectra compared with literature data and by acid hydrolysis followed by GC analysis of the corresponding trimethylsilylated monosaccharides, Puriﬁed saponin 1 (the structure was established as *3β-O-*{*β-D-xylopyranosyl-(1→3*)*- α-L-rhamnopyranosyl-(1→2*)*-α-L-arabinopyranosyl*} *oleanolic acid* as shown in Figure 1C. Saponin 1 was prepared by dissolving it in dimethylsulfoxide (DMSO) (Gibco BRL, Invirtogen, Carlsbad, CA) followed by further dilution in fresh tissue culture medium. In all the experiments, the final DMSO concentration did not exceed 1‰ (v/v), so as not to affect cell growth. Cells incubated with saponin 1-free culture medium which contains 1‰ DMSO were used as vehicle-controls.

### Ethics statement

Primary cultured astrocytes were prepared from non-neoplastic brain parenchyma from an informed and consenting volunteer with cerebral trauma (consented by ethics committee from Xijing Hospital, the Fourth Military Medicine University; XJYYLL-2011207). The volunteer received neurosurgery in our institution under the approval of the local medical research ethics committee.

### Cell lines and cell culture

Human glioblastoma U251MG and U87MG cells (obtained from a cell bank at the Fourth Military Medical University, China) were cultured in DMEM medium supplemented with 10% newborn calf serum (GIBCO BRL, Invitrogen) in a 37 °C incubator with a humidiﬁed atmosphere of 5% CO_2_ and 95% O_2_. Twenty-four hours before each experiment, cells were transferred to serum free medium. Saponin 1 at indicated concentrations was added to the culture medium. Saponin 1-free DMEM medium which contains 1‰ DMSO were used as vehicle-controls.

### MTT assay

Loss of cell viability was determined by the 3-(4, 5-dimethylthiazol-2-yl)-2, 5-diphenyltetrazolium bromide (MTT) assay as described previously [12]. MTT was added to the cells at a ﬁnal concentration of 5 mg/ml and incubated for 4 h, allowing the reduction in MTT to produce water-insoluble dark blue formazan crystals. Media was then removed and cells were dissolved in DMSO. Formazan production was measured by the absorbency change at 490 nm using a microplate reader (BioRad Laboratories, Hercules. CA). Viability results were expressed as percentages. The absorbency measured from saponin 1-free DMEM-incubated cells was set at 100%.

### Hoechst 33342 staining

Hoechst 33342 staining was carried out to detect apoptotic nuclei. Primary cultured astrocytes and human glioblastoma U251MG and U87MG cells were grown in 6-well plates and treated with saponin 1 (7.4 µg/ml ) for 24 h or in the presence of saponin 1-free culture medium. After washing with phosphate buffered saline (PBS, 0.01 M, pH 7.4) and fixing the cells in 70% ethanol for 2 h at 4°C, cells were incubated for 3 min with a solution of Hoechst 33342 in PBS. After a final wash in PBS, nuclear morphology changes were visualized by fluorescence microscopy (Leica Microsystems, Wetzlar, Germany) using excitation wavelengths between 330 and 380 nm. Digitized images were captured.

### Electron microscopy

Primary cultured astrocytes and human glioblastoma U251MG and U87MG cells were cultured in T-150 ﬂasks (Greiner BioOne GmbH, Frickenhausen, Germany) (3 × 10^6^ cells/cm^2^) and treated with saponin 1 (7.4 µg/ml ) for 24 h. Then, the cells were trypsinized with 0.25% trypsin and centrifuged at 1,400 g for 15 min. The pellets were ﬁxed and embedded for transmission electron microscopy according to procedures described previously [13,14]. Thin sections (75 microns) were cut on an ultramicrotome and double stained with uranyl acetate and lead citrate. Electron micrographs were taken on an electron microscope (JEM-2000EX, JEOL Ltd., Tokyo, Japan) operating at 80 kV.

### Apoptosis-DNA ladder assay

DNA was isolated from primary cultured astrocytes and human glioblastoma U251MG and U87MG cells treated with 7.4 µg/ml saponin 1 for 24 h using a DNeasy Tissue Kit (QIAGEN, Inc., Mississauga, ON). The isolated DNA was resolved on a 1.5% agarose gel containing ethidium bromide in 40 mM Tris-acetate buffer (pH 7.5) with electrophoresis at 50 V for 4 h. DNA fragments were photographed under UV light.

### Flow cytometry for Annexin V/propidium iodide (PI) staining

To determine the number of apoptotic cells, Annexin V assays were performed using an apoptosis detection kit (Annexin V-FITC/PI Staining Kit; Immunotech Co., Marseille, France). Brieﬂy, cells were plated onto 60-mm culture dishes at a density of 2 × 10^5^ cells per dish and treated with 7.4 µg/ml saponin 1 for 24 h. Cells were harvested and washed in cold PBS, and then incubated for 15 min with ﬂuorescein-conjugated AnnexinV and PI. Then, the cells were analyzed using ﬂow cytometery and Modﬁt software (Verity Software House, Inc., Topsham, ME). Cells in the right lower quadrant of the density dot plots from the flow cytometric analysis represented early apoptotic cells, while cells in the left low quadrant, which were both PI and AnnexinV negative, were considered normal.

### Immunocytochemistry

Immunocytochemistry and evaluation of the immunoreactivity scores (IRS) for nuclear NF-κB p65 were performed as previously described [15]. Primary cultured astrocytes and glioblastoma U251MG and U87MG cells were cultured on glass slides and treated with or without 7.4µg/ml saponin 1 for 24 h, respectively. Cells were stained with a primary anti- NF-κB p65 mouse monoclonal antibody (1:50) followed by a biotinylated goat anti-mouse IgG (1:50) secondary antibody. Both antibodies were purchased from Santa Cruz Biotechnology (Santa Cruz, CA).

### Western blot analysis

Astrocytes and glioblastoma, U251MG and U87MG cells treated with 7.4 µg/ml saponin 1 for 12, 24, and 72 h, respectively, were prepared in RIPA buffer (150Mm, NaCl, 1% NP-40, 0.5% sodium deoxycholate, 0.1% SDS, 50mM Tris-HCl, pH 8.0), 10mM EDTA and 1 mM PMSF (Sigma, Chemical, St. Louis, MO) for 30 min at 4°C. Samples were then centrifuged for 20 min at 14,000g. Protein concentrations were determined using the BCA protein Assay Kit (Pierce, Rockford, IL). Equivalent amounts (25 ug) of protein lysates were separated by 10% SDS-PAGE and transferred to nitrocellulose membranes (0.22 μm, Millipore, MA, USA). Membranes were blocked for 2 h at room temperature with blocking buffer (TBS containing 0.1% Tween-20 [w/v]) and 5% milk (w/v). Primary antibodies were applied for 1 h at room temperature or overnight at 4°C as appropriate. The following primary antibodies were used: anti-p65 (1:300, mouse monoclonal), anti-Survivin (1:300, mouse monoclonal), anti-XIAP (1:300, rabbit polyclonal), anti-Bax (1:600, rabbit polyclonal), anti-Bcl-2 (1:600, rabbit polyclonal), anti-cleaved caspase-9 (1:100, mouse monoclonal), anti-cleaved caspase-3 p11 (1:150, rabbit polyclonal) and anti-β-actin (1:1000, mouse monoclonal). The following secondary antibodies were used: HRP-conjugated anti-rabbit IgG (1:2000) and HRP-conjugated anti-mouse IgG (1:2000). All antibodies were purchased from Santa Cruz Biotechnology. The band intensities were quantified using the image analysis software ImageJ (http://rsb.info.nih.gov/ij/index.html). The integrated density of each band was normalized to the corresponding human β-actin band.

### Cell fractionation and western blot analysis for nuclear-located NF-kappaB

Cells were fractionated using the Qproteome Nuclear Protein Kit (No. 37582) to generate cytosolic and nuclear pools as described by the manufacturer (Qiagen). After quantified, equivalent amountsof cytosolic (25 ug) and nuclear (30 ug) protein lysates were determined for NF-kappaB expression respectively by western blotting. Anti-p65 (1:300, mouse monoclonal) and anti-Lamin B (1:400, mouse monoclonal) were used as primary antibodies.

### In vivo experiment

A total of 32 female BALB/c-nu/nu mice weighing 15-18 g and 5 weeks of age were purchased from the Shanghai SLAC Laboratory Animal Company, Ltd. The nude mice were maintained in pathogen-free conditions at 26°C, at 70% relative humidity and under a 12-hr light/dark cycle. All animal experiments complied with the international guidelines for the care and treatment of laboratory animals under the approval of local ethics committee of our university. The mice were divided randomly into two saponin-1-treated groups (U87MG+ and U251MG+ groups) and two vehicle-control groups (U87MG- and U251MG- groups). Briefly, the cells (1×10^8^) were suspended in 0.2 mL of normal saline in each group, and then were inoculated subcutaneously into the right flank of nude mice. After the development of palpable nodules, mice were treated with 10 µg/mL×200µL saponin 1 every three days by injection via tail veins in the two treatment groups. By contrast, mice in the two vehicle-control groups were injected with normal saline (contains 1‰ DMSO) in equal volume (200µL). Tumor volumes in mice were measured with a slide caliper every five days until the scarification, which was performed 30 days after inoculation of tumor cells, and recorded using the formula: volume=a×b^2^/2, where “a” stands for the larger, whereas “b” stands for the smaller of the two dimensions.

### Statistical analysis

Statistical comparisons were performed using a Student’s t-test or one-way analysis of variance (ANOVA) followed by a Bonferroni multiple comparisons test with the Instat statistics program (GraphPad Software Inc., San Diego, CA). P > 0.05 was considered statistically significant. All the above mentioned experiments were repeated in triplicate.

## Results

### Saponin 1 suppressed the cell viability of glioblastoma cells

**Figure 2:**
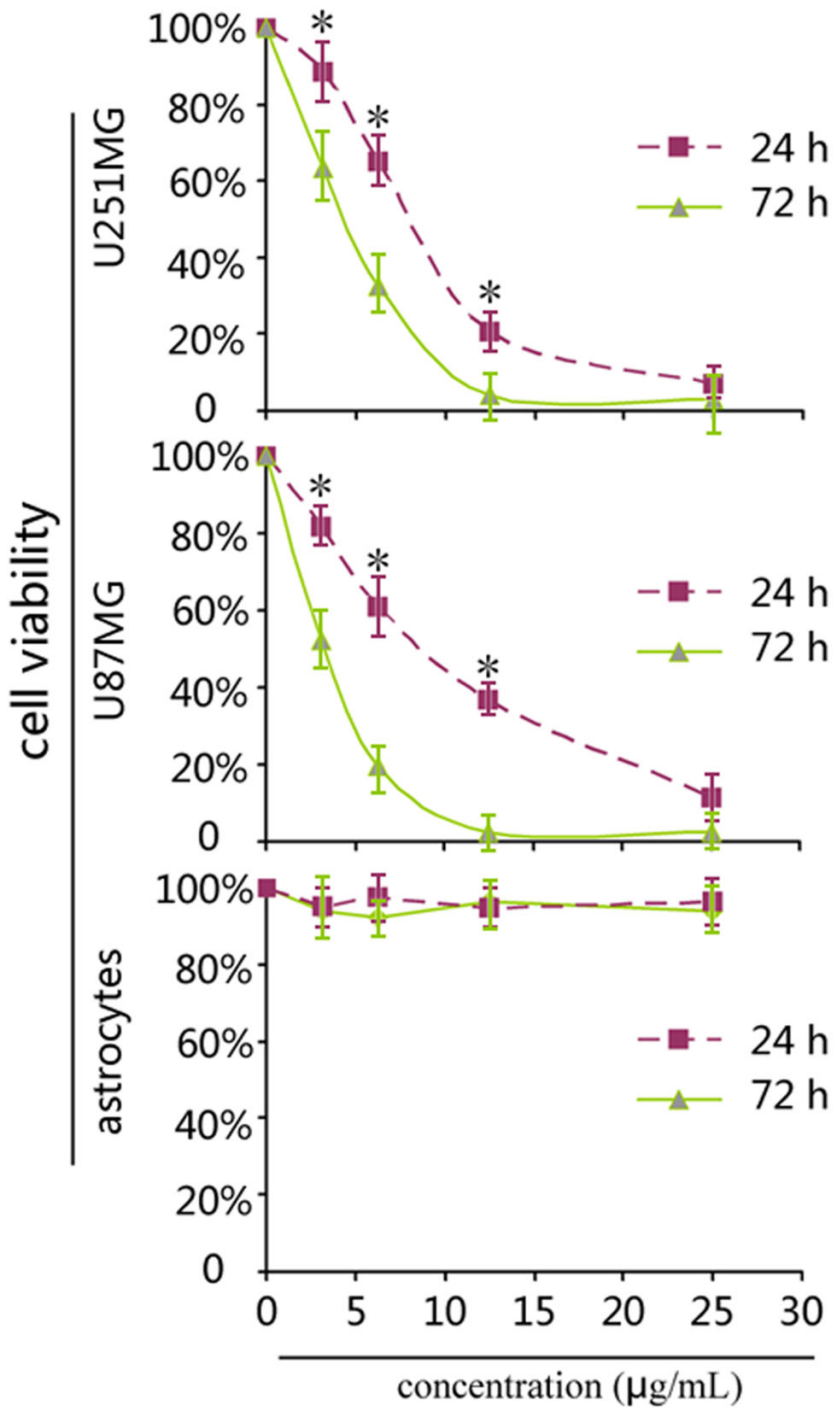
MTT assay. Saponin 1 significantly inhibited the cell viabilities of glioblastoma U87MG and U251MG cells in a dose concentration- and time-dependent manner, but did not affect the cell viability of primary cultured astrocytes, as compared to the vehicle-controls.

To evaluate the cytotoxic effect of saponin 1 on glioblastoma U251MG and U87MG cells, cells were treated with saponin 1 at uniform-gradient concentrations followed by cell viability measurements using the MTT assay at 24 h and 72 h, respectively. The cellular proliferation of glioblastoma U251MG and U87MG cells was significantly decreased following saponin 1 treatment in a dose- and time-dependent manner. The inhibitory effects of saponin 1 were similar between the two cell lines. Growth inhibition of saponin 1 was more prominent in U87MG cells than in U251MG cells. Saponin 1 (10 µg/ml) treatment for 24 h markedly decreased the cell viability of U251MG and U87MG cells to 28.6±0.4% and 42.5±0.6%, respectively, when compared to the vehicle-treated controls (Figure 2). The growth inhibitory dose of 50% (ID50) of saponin 1 (cells were treated for 24 h) was 7.4 µg/ml in U251MG cells and 8.6 µg/ml in U87MG cells. Furthermore, ID50 of saponin 1 was less than 5 µg/ml in both glioblastoma cell lines which were treated for 72 h. In addition, saponin 1 treatment did not affect the cell viability of primary cultured astrocytes. Results suggested that the cell viability of primary cultured astrocytes treated with 20 µg/ml saponin 1 for 72 h was minimally affected.

### Saponin 1 induced apoptosis in glioblastoma cells

**Figure 3:**
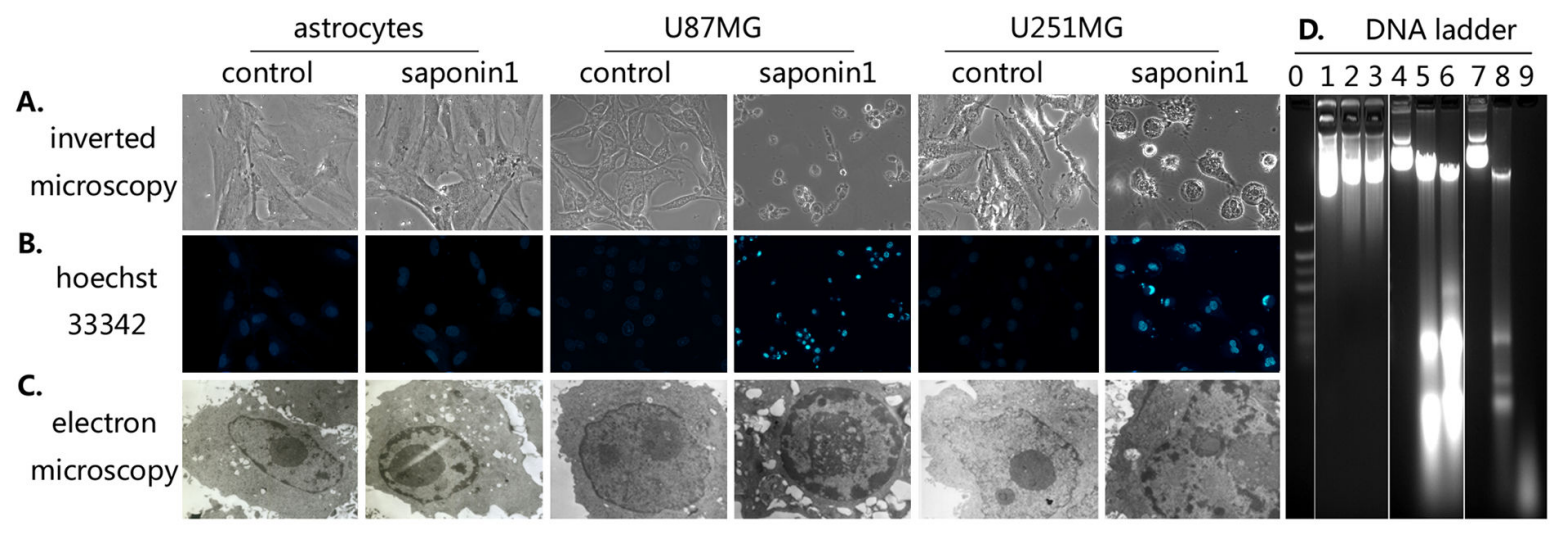
Saponin 1 treatment resulted in significant apoptotic morphological changes in glioblastoma U87MG and U251MG cells. A, inverted microscopic observation. B, Nuclear fluorescent Hoechst 33342 staining. C, electron microscopic observation. D, electrophoresis of cellular DNA, lane 0, marker; lane 1-3, primary cultured astrocytes exposed to 7.4 μg/mL saponin-1 for 0, 24, and 72 hours; lane 4-6, U87MG cells exposed to 7.4 μg/mL saponin-1 for 0, 24, and 72 hours; lane 7-9, U251MG cells exposed to 7.4 μg/mL saponin-1 for 0, 24, and 72 hours.

**Figure 4:**
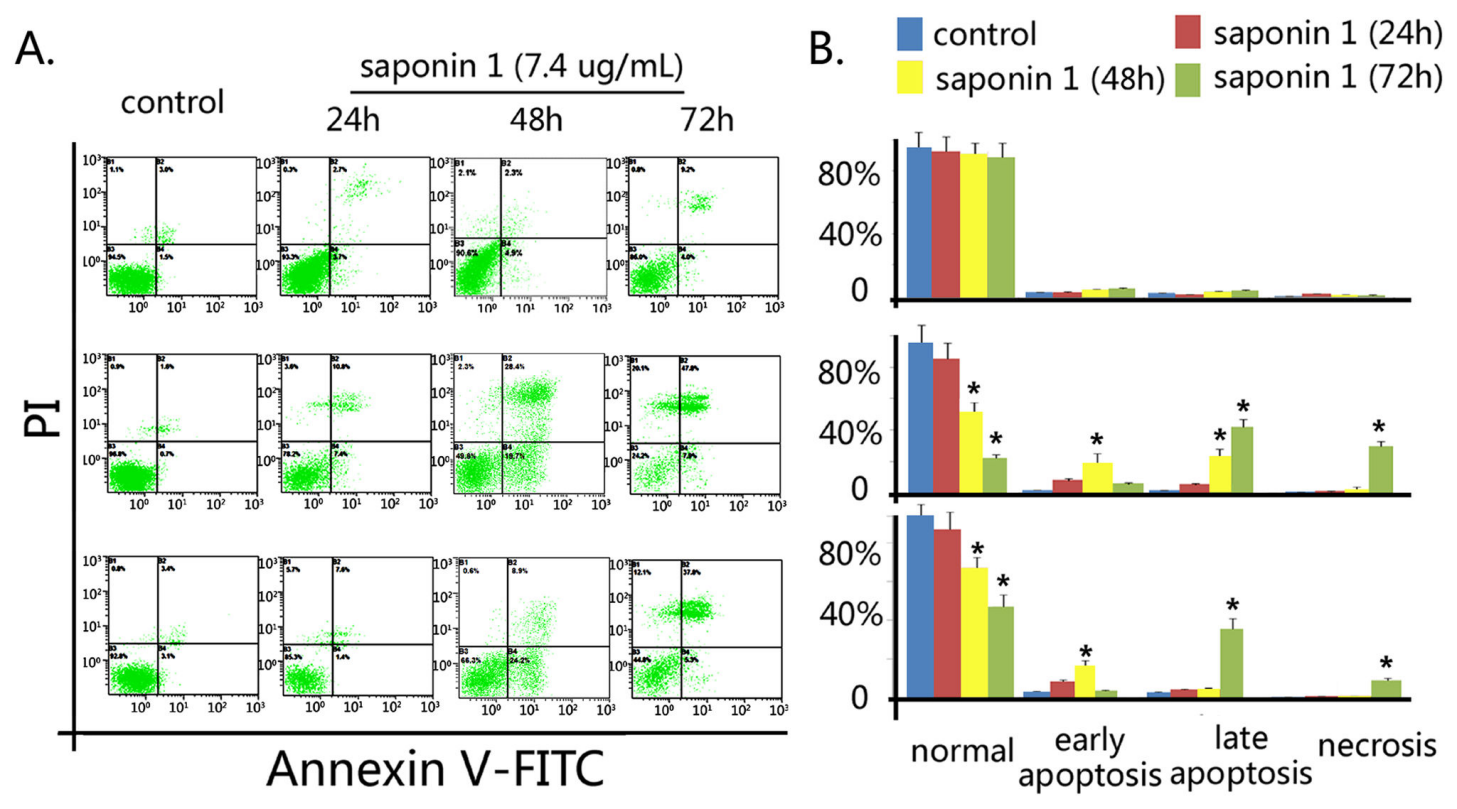
AnnexinV/PI-based flow cytometry. Semiquantitative AnnexinV/PI data suggested that saponin 1 significantly induced apoptosis and necrosis in a time-dependent manner in glioblastoma U87MG and U251MG cells, but not in primary cultured astrocytes.

In contrast to the normal morphological features of primary cultured astrocytes treated with saponin 1, microscopic observation of glioblastoma U251MG and U87MG cells indicated that apoptosis accounted for the inhibitory effect of saponin 1 (Figure 3). Gioblastoma cells showed characteristic morphological features such as cell shrinkage, aggregation, and detachment from the surface of the culture ﬂask (Figure 3A). Hoechst 33342 staining showed that glioblastoma cell nuclei had notable condensation and eventual fragmentation (Figure 3B). In addition, electron microscopy revealed intracellular structure apoptotic change, including swelling of mitochondria, loss of microvilli, and abundant formation of lysosomes (Figure 3C). Electrophoresis of cellular DNA revealed that saponin 1 induced apoptosis-speciﬁc DNA cleavage in glioblastoma cells, as evidenced by high levels of a DNA fragmentation (Figure 3D). These morphological observations were further confirmed by semi quantitative AnnexinV/PI analyses (Figure 4A and 4B). Following treatment of saponin 1 (7.4 µg/ml ) for 24 h and 72 h, the percentage of apoptotic cells was 12.2 ± 0.4% and 44.5 ± 0.3% in U251MG cells and 14.2 ± 0.5% and 47.6 ± 0.5% in U87MG cells, respectively. In addition, saponin 1 induced greater necrosis in U87MG cells than that in U251MG cells at 72 h (28.9 ± 0.8% vs. 8.5 ± 0.6%, p = 0.038).

### Saponin 1 suppressed the intracellular expression and nuclear translocation of NF-κB in glioblastoma cells

**Figure 5:**
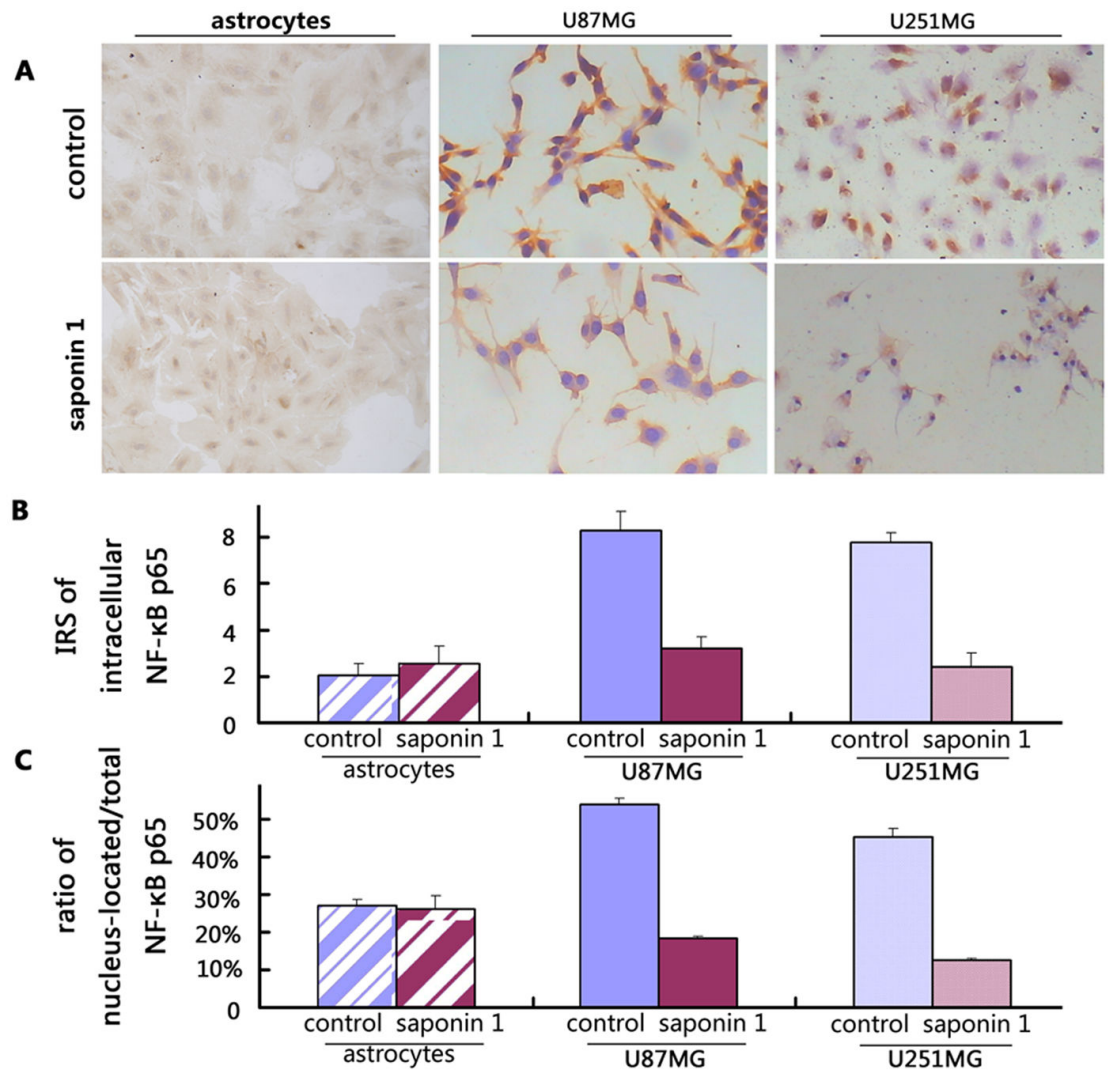
NF-κB p65-specific immunocytochemistry in glioblastoma U87MG and U251MG cells. A, representative immunocytochemical photos targeting NF-κB p65 in primary cultured astrocytes and glioblastoma cells. B, IRS scoring of intracellular expression of NF-κB p65. C, IRS scoring of nuclear NF-κB p65.

**Figure 6:**
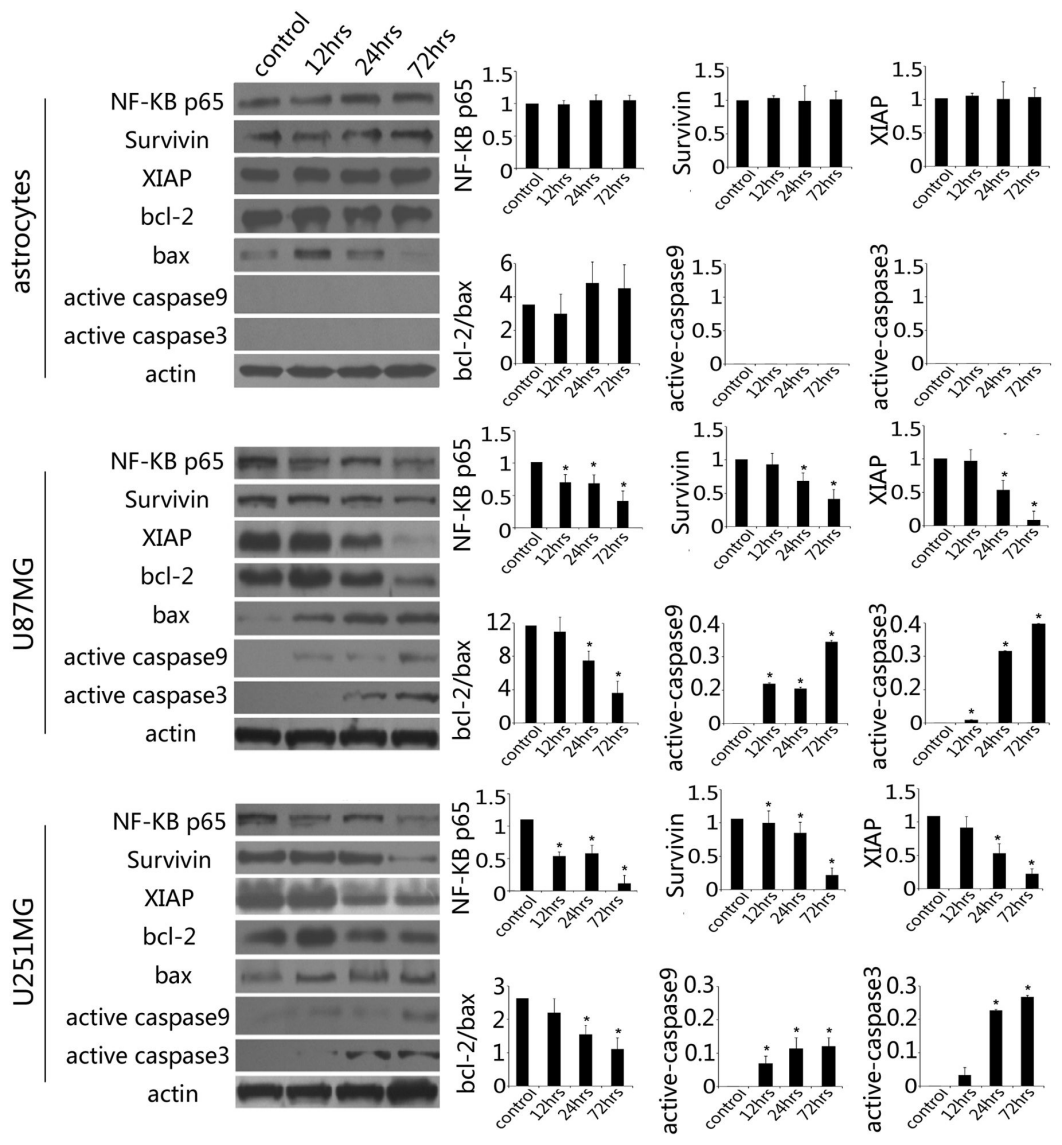
Western blotting analysis. Saponin 1 treatment resulted in a time-dependent decrease in the expressions of NF-κB p65, survivin and XIAP in glioblastoma cell lines. The ratio of Bcl-2/Bax rapidly diminished and the activation of caspase-9 and caspase-3 was observed. Endogenous expression of these proteins was not changed in primary cultured astrocytes exposed to saponin-1-treatment.

**Figure 7:**
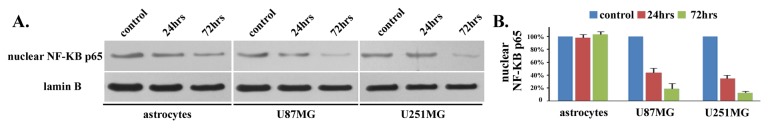
Nuclear NF-κB p65 expression. A. Nuclear expression of NF-κB p65 was obviously down-regulated in a time-dependent manner in both U87MG and U251MG cells. In contrast, it was not affected in primarily cultured astrocytes. B. statistical histogram.

To investigate the possible involvement of pro-survival NF-κB signaling as part of the anti-cancer properties of saponin 1 in glioblastoma cells, we performed immunocytochemistry. Our results demonstrated that the intracellular expression of NF-κB p65 was significantly down-regulated in saponin 1-treated glioblastoma cell lines compared to vehicle-treated controls. According to the immunocytochemical results, which were interpreted by two independent neuropathologists, the immunoreactivity score of intracellular NF-κB p65 was 7.8 ± 0.4 and 8.3 ± 0.8 in vehicle-treated U251MG and U87MG cells, respectively. These scores decreased to 2.4 ± 0.6 and 3.2 ± 0.5 in U251MG and U87MG cells when exposed to 7.4 µg/ml saponin 1 for 24 h, respectively (Figure 5A). Moreover, after the same treatment schedule, the ratio of nucleus-located to total NF-κB p65 reduced from 45.2 ± 2.3% to 12.5 ± 0.5% in U251MG cells and from 54.0 ± 1.6% to 18.3 ± 0.7% in U87MG cells (Figure 5B). Western blotting showed slight repression of endogenous NF-κB p65 was observed in both glioblastoma cell lines following treatment of 7.4 µg/ml saponin 1 for 4 h (data not shown). As shown in Figure 6, saponin 1 led to a 56.2 ± 4.5%, 68.0 ± 5.2% and 83.7 ± 5.8% reduction of NF-κB p65 expression in U251MG cells at 12 h, 24 h and 72 h, respectively. Western blot analysis also indicated a similar time-dependent reduction in NF-κB p65 expression in U87MG cells of 48.3 ± 3.8%, 58.6 ± 6.8%, and 84.8 ± 4.5% following saponin 1 treatment for 12 h, 24 h, and 72 h, respectively. Furthermore, the nuclear NF-κB p65 expressions were markedly repressed by saponin-1 treatment as shown in Figure 7. It suggested that nuclear NF-κB p65 expression of U87MG and U251MG cells decreased to 42.6 ± 3.2% and 31.5 ± 2.7% respectively after saponin 1 treatment for 24 h, and 20.7 ± 4.2% and 11.2 ± 2.4% respectively after saponin 1 treatment for 72 h, compared to their vehicle-controls.

In contrast, saponin 1 did not affect the expression and nuclear translocation of NF-κB in primary cultured astrocytes. Immunocytochemistry showed a moderate expression of NF-κB in primary cultured astrocyte, with a score of 2.0 ± 0.4 and 2.2 ± 0.6 before and after saponin 1 treatment, respectively. Meanwhile, the ratio of nucleus-located to total NF-κB p65 was statistically unchanged upon saponin 1 treatment (37.5 ± 4.2% vs. 36.3 ± 7.4%, p>0.05).

### Saponin 1 decreased the levels of IAPs

In earlier experiments, we demonstrated that saponin 1 treatment (7.4 µg/ml) exhibited moderate suppression of survivin and XIAP as early as 6 h following treatment (data not shown). Twenty-four hours following treatment, survivin levels were down-regulated by 42.4 ± 2.8% and 38.0 ± 4.5% in U251MG and U87MG cells, respectively, compared with the vehicle controls (Figure 6B and 6C). Moreover, the suppressive effect of saponin 1 on survivin expression occurred at 72 hours. 

As shown in Figure 6, incubation with saponin 1 for 24 hours decreased XIAP expression to 34.6 ± 4.8 % and 25.0 ± 8.2% in U251MG and U87MG cells, respectively, when compared with the vehicle controls. Interestinly, XIAP expression in U87MG cells became negligible at 72 hours, whereas XIAP expression in U251MG cells did not change following 24 h and was present at a low level.

In contrast, western blot showed that survivin and XIAP proteins in saponin 1-treated primary cultured astrocytes did not significantly change (Figure 6A).

### Saponin 1 mediated the down-regulation of Bcl-2 expression and up-regulation of Bax expression in glioblastoma cells

The ratio of Bcl-2/Bax is critical for the induction of apoptosis, especially in the classic apoptotic intrinsic pathway. Western blot showed that the endogenous expression of Bcl-2 was robust in both glioblastoma cell lines, while Bax expression was very weak. As shown in Figure 6, saponin 1 treatment significantly induced Bax expression and inhibited Bcl-2 expression in both glioblastoma cell lines (38.5 ± 4.8% and 27.1 ± 2.5% in U251MG and U87MG cells, respectively), resulting in a decreased Bcl-2/Bax ratio when compared with the vehicle controls (Figure 6B and 6C). The ratio of Bcl-2/Bax in primary cultured astrocytes did not change following saponin 1 treatment (Figure 6A).

### Saponin 1 stimulated the activation of caspase-9 and caspase-3

Activation of the caspase cascade is the hallmark of apoptosis. To determine whether saponin 1 was associated with the cleavage and activation of caspase enzymes, cleaved caspase-9 and caspase -3 expressions were investigated in saponin 1-treated glioblastoma cells and primary cultured astrocytes (Figure 6). Capase-9 and -3 were not activated in primary cultured astrocytes. However, Western blot showed that activation of caspase-9 by saponin 1 occurred as early as 12 h, and persistently increased at all time-points, in both of the glioblastoma cell lines. Saponin 1 treatment also led to significant caspase-3 activation subsequent to caspase-9 activation. Cleaved caspase-3 expression became detectable following saponin 1 treatment for 18 h and 12 h in U251MG and U87MG cells, respectively, and was augmented gradually in a time-dependent manner. 

### Saponin 1 suppressed the tumor growth in vivo

**Figure 8:**
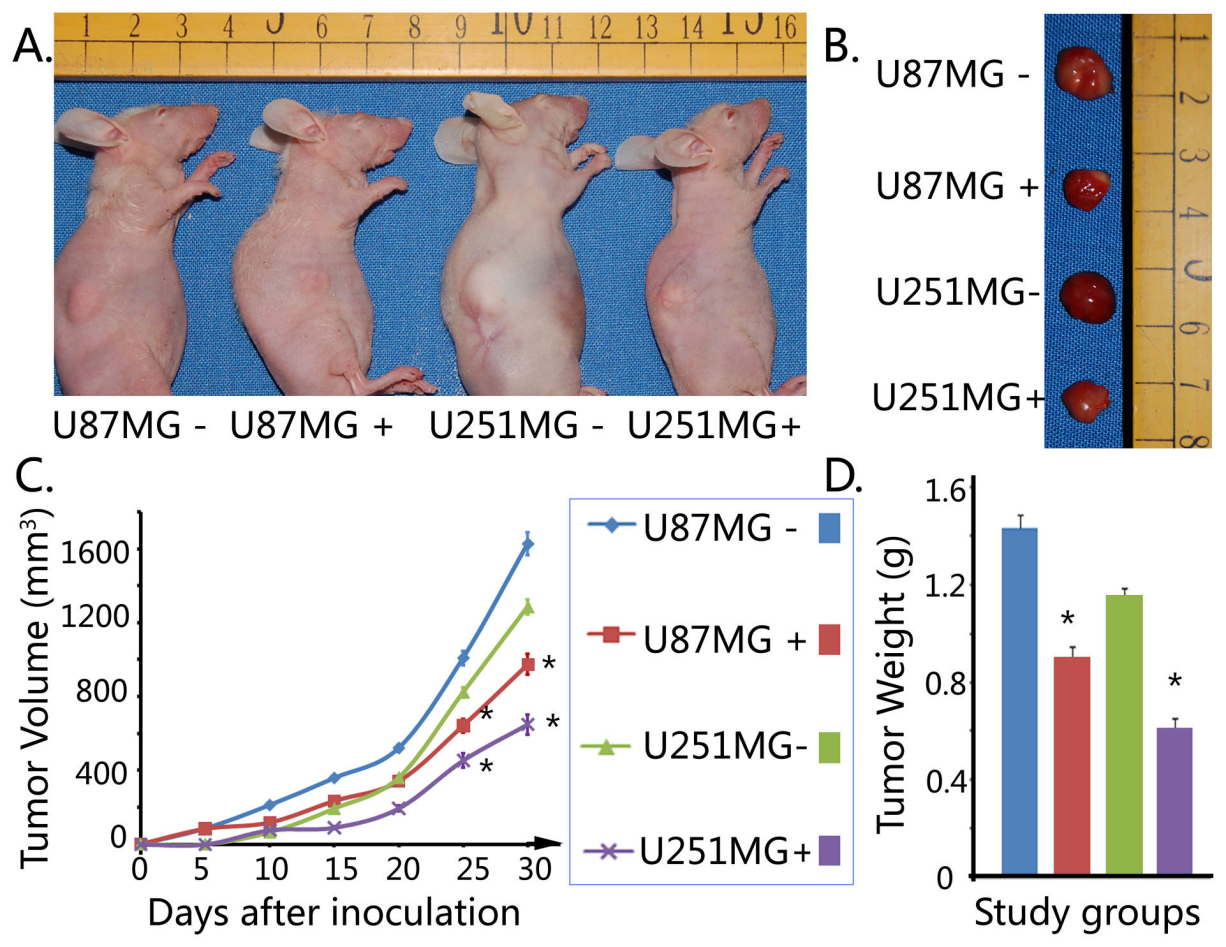
*In vivo* anti-tumor effect of saponin 1 in nude mice. Saponin 1 significantly inhibited tumor growth of U87MG and U251MG cells-produced xenograft tumors. A and B. Typical features of tumoral nodules in nude mice. C. Increase in tumor volume was makedly inhibited in saponin 1-treated nude mice. D. Tumor weight was markedly down-regulated in saponin 1-treated nude mice.

To investigate the inhibitory effect of saponin 1 on glioblastoma cells in vivo, we tested the tumor growth of xenograft tumor produced by both glioblastoma cell lines exposing to 10 µg/ml saponin 1. Xenograft tumor of U87MG cells showed faster tumorigenesis and tumor growth, and was more sensitivity to saponin 1 treatment, compared with those of U251MG cells. Palpable nodules were developed in nude mice implanted with U87MG and U251MG cells 4-5 days and 8-10 days after inoculation, respectively. In addition, the tumor growth was markedly suppressed in nude mice from both two treatment groups after injection of saponin 1. By contrast, tumors in the two control groups persistently grew in an accelerative fashion. At the time of scarification, the average volumes of tumors in U251MG- group, U251MG+ group, U87MG- group, and U87MG+ group were 1628±62 mm^3^, 974±56 mm^3^, 1286±41 mm^3^, and 648±53 mm^3^, respectively. The typical features and the detailed parameters of xenograft tumors in the four study groups were shown in Figure 8.

## Discussion

Treatment of glioblastoma remains a major clinical challenge in neuro-oncology due to difficulties in resection. Therefore, the glioblastoma treatment usually consists of a combination of surgery, radiation therapy, and chemotherapy. Although the clinical implications chemotherapeutic agents such as temozolomide and irinotecan have shown improvements in regard to overall survival and quality of life, accumulating evidence suggests that nearly 50% of patients do not benefit from these drugs due to cellular resistance to chemotherapeutic agents, as well as O-6-methylguanine-DNA methyltransferase (MGMT) content in glioblastoma cells [16]. In the current study, we investigated the anti-neoplastic effectiveness of saponin 1 in human glioblastoma U251MG and U87MG cells. We found that saponin 1 treatment significantly inhibited cell growth and induced apoptosis in glioblastoma cells. Our results showed that the inhibitory effect of saponin 1 on cell proliferation was associated with DNA damage, increased phosphatidylserine exposure, inactivation of NF-κB, and the down-regulation of survivin and XIAP expression. Furthermore, the Bcl-2/Bax ratio was decrease and caspase cascade was initiated, suggesting that saponin 1 induced apoptosis in glioblastoma cells.

In this study, saponin 1 appeared to be well tolerated in normal cells *in vitro*, as indicated by the MTT assay. Specifically, the cellular viability of primary cultured astrocytes was minimally affected following saponin 1 treatment. Since astrocytes are the major component of brain parenchyma, the MTT data suggests that saponin 1 therapy may play a treatment role in GBM with minimal toxicity to normal cells. Although the mechanisms underlying the differential sensitivity to saponin 1 in glioblastoma cell lines and primary astrocytes was not been fully elucidated in this study, current studies suggest that unlike normal cells, mitosis as well as nuclear duplication in cancer cells is more frequent and conspicuous. Hence, cancer cells are more likely to be destroyed by cytotoxic drugs that are usually targeted in mitosis-associated molecules. However, the systematic safety profile of saponin 1 must be established with regard to the cytotoxicity on neurons and vascular endothelial cells, and the potential risk on other organs should be strictly determined in subsequent animal experiments and clinical trials.

In this study, saponin 1 induced notably early apoptotic events in glioblastoma U251MG and U87MG cells. Apoptosis, or programmed cell death, is the principle mechanism by which chemotherapeutic agents kill cancer cells. Our results are consistent with previous studies evaluating saponin agents. Apoptosis immediately occurred when glioblastoma cells were exposed to saponin 1 at low concentrations, suggesting a rapid pharmacologic action of saponin 1, providing an experimental basis for quick and effective chemotherapy. In addition, our current study found that NF-κB-mediated survival signals were differentially regulated by saponin 1 treatment in primary cultured astrocytes and glioblastoma cells. The expression and nuclear translocation of NF-κB, endogenous expression of survivin and XIAP proteins, the ratio of Bcl-2/Bax, and the activation of caspase-9 and caspase-3 were rarely affected by saponin 1 treatment in primary cultured astrocytes. In contrast, there were striking changes that occurred in both the glioblastoma cell lines. In our preliminary study to investigate the possible molecular mechanisms underlying the pro-apoptotic bioactivity of saponin-1, our data revealed that modulations of NF-κB, survivin and XIAP proteins occurred at the early stage of saponin-1-induced apoptosis, which preceded the activation of capsase-9 and caspase-3. Interestingly, the expression and nuclear translocation of NF-κB was the earliest molecular feature that accompanied growth inhibition, phosphatidylserine exposure, DNA cleavage, and apoptotic morphological alterations. These results suggest that the repression of NF-κB-mediated survival signaling may be partially responsible for the anti-tumor activity of saponin-1.

Constitutive activation of proteins in the NF-κB signaling pathway is an important genetic feature of glioblastoma cells [17]. NF-κB is a key mediator of survival signaling and is responsible for the transactivation of various target genes that are implicated in cell survival, decreased apoptosis and increased cell growth [18]. Studies have shown that the presence of NF-κB in the nucleus is critical for the maintenance of a malignant phenotype of glioblastoma cells [19] and is an unfavorable prognostic factor that impacts the long-term survival of glioblastoma patients [20]. A recent study demonstrated that inhibition of NF-κB with bortezomib, proteasome inhibitor, enhanced the anti-tumor effects of docetaxel [21], which could lead to improved treatment outcomes by reducing chemoresistance. In our current study, from immunocytochemistry and Western blot data supported our hypothesis that initiation of apoptosis induced by saponin 1 was associated with the down-regulation and inactivation of NF-κB.

In addition, IAP family members, such as survivin and XIAP, are involved in another pro-survival signaling pathway that is involved in the resistance of pro-apoptotic signals induced by chemotherapeutic agents [22]. Inhibition of IAP family member expression has been shown to result in cell death in some glioblastoma cells [23,24]. Aberrant expression of the survivin protein in glioblastoma specimens and its prognostic significance to identify patients with poor overall survival has been described in a previous study [25]. It is suggested that survivin expression increases gradually according to the pathological grades of glioma specimens and is much more abundant in glioblastomas than those in low-grade gliomas [26]. Moreover, survivin expression was found to be inversely correlated with spontaneous apoptosis in glioblastoma cells, suggesting that it could be a potential target for molecular therapy [27]. Ongoing investigations conducted by other groups have expanded the understanding of the possible role of survivin in the chemoresistance of glioblastomas and other cancers [28]. These findings suggest that inhibition of survivin contributes to defects in cell division and induces apoptosis via pro-apoptotic Bcl-2 family members, resulting in the subsequent release of cytochrome c, depolarization of the mitochondrial outer membrane, and the eventual activation of the caspase cascade [29]. In our present study, we found that the inhibition of survivin was associated with saponin 1-induced caspase activation and glioblastoma cell apoptosis, which was consistent with previous studies.

In conclusion, saponin 1 exhibited a dose- and time-dependent inhibition of cellular growth and activation of apoptosis in the glioblastoma U251MG and U87MG cell lines. The anti-glioblastoma activity of saponin 1 was characterized by a significant inhibition of NF-κB with a subsequent down-regulation of survivin and XIAP. Saponin 1 also increased the cellular content of pro-apoptotic Bax protein and led to the activation of caspase-9 and caspase-3. Further *in vivo* studies are needed to validate the role of saponin 1 as a new agent for the treatment of chemoprevention of glioblastoma.

## Supporting Information

Table S1 **^13^C-NMR (125 MHz) data of saponin 1 (in pyridine-d_5_).**
(DOC)
