# Correction: Saponin 1 Induces Apoptosis and Suppresses NF-κB-Mediated Survival Signaling in Glioblastoma Multiforme (GBM)

**DOI:** 10.1371/annotation/0c3019ff-6d7b-444b-8eb5-9461f3dcd029

**Published:** 2014-01-10

**Authors:** Juan Li, Haifeng Tang, Yun Zhang, Chi Tang, Bo Li, Yuangang Wang, Zhenhui Gao, Peng Luo, Anan Yin, Xiaoyang Wang, Guang Cheng, Zhou Fei

There is an error in affiliation for the corresponding author, Dr. Guang Cheng. The correct affiliation is: Department of Neurosurgery, Xijing Institute of Clinical Neuroscience, Xijing Hospital, Fourth Military Medical University, Xi’an, China.

