# Supplementary material for: Saponin 1 Induces Apoptosis and Suppresses NF-κB-Mediated Survival Signaling in Glioblastoma Multiforme (GBM)
Source: PLoS One. 2013 Nov 21;8(11):e81258. doi: 10.1371/journal.pone.0081258 (PMC3836797; doi:10.1371/journal.pone.0081258)
Supplement: Table S1 — 13C-NMR (125 MHz) data of saponin 1 (in pyridine-d5). (DOC) [file pone.0081258.s001.doc]

**Table** S**1.** ^13^C-NMR (125 MHz) data of saponin 1 (in pyridine-*d*_5_).

| C | **1** |  | C | **1** |
| --- | --- | --- | --- | --- |
| 1 | 38.8 |  | 3-*O*-sugar | |
| 2 | 26.6 |  | Ara |  |
| 3 | 88.7 |  | 1 | 105.2 |
| 4 | 39.7 |  | 2 | 75.6 |
| 5 | 55.9 |  | 3 | 74.6 |
| 6 | 18.5 |  | 4 | 69.3 |
| 7 | 33.1 |  | 5 | 65.6 |
| 8 | 39.5 |  | Rha |  |
| 9 | 48.0 |  | 1 | 101.4 |
| 10 | 37.0 |  | 2 | 71.9 |
| 11 | 23.7 |  | 3 | 82.9 |
| 12 | 122.4 |  | 4 | 72.9 |
| 13 | 144.9 |  | 5 | 69.6 |
| 14 | 42.1 |  | 6 | 18.4 |
| 15 | 28.3 |  | Xyl |  |
| 16 | 23.7 |  | 1 | 107.4 |
| 17 | 46.7 |  | 2 | 75.3 |
| 18 | 42.0 |  | 3 | 78.4 |
| 19 | 46.5 |  | 4 | 71.1 |
| 20 | 30.9 |  | 5 | 67.4 |
| 21 | 34.2 |  | Glc |  |
| 22 | 33.2 |  | 1 |  |
| 23 | 28.1 |  | 2 |  |
| 24 | 17.1 |  | 3 |  |
| 25 | 15.5 |  | 4 |  |
| 26 | 17.4 |  | 5 |  |
| 27 | 26.1 |  | 6 |  |
| 28 | 180.4 |  | 28-*O*-sugar | |
| 29 | 33.3 |  | Glc |  |
| 30 | 23.7 |  | 1 |  |
|  |  |  | 2  3  4  5  6 |  |

Physical and spectroscopic data of compounds

Saponin 1: White amorphous powder; m. p. 232~235 °C; [*α*]Combin +1.6 (*c* 0.13, MeOH) (supplementary materials: Table **1)**; ESI-MS (pos. ion mode) *m/z* 889 [M+Na]^+^; ESI-MS (neg. ion mode) *m/z* 865 [M−H]^−^; ESI-MS/MS (neg. ion mode, parent ion at *m/z* 865) *m/z* 733 [865−132]^−^, 587 [733−146]^−^, 455 [587−132]^−^. ^1^H-NMR (500 MHz, pyridine-*d*_5_) *δ*: 0.82, 0.94, 0.97, 0.99, 1.12 (each 3H, s, CH_3_), 1.29 (6H, s, 2×CH_3_) , 1.53 (3H, d, *J*=6.0 Hz, CH_3_ of rha), 3.27 (1H, m, H-3), 3.29 (1H, m, H-18), 4.84 (1H, d, *J*=6.0 Hz, H-1 of ara), 5.33 (1H, d, *J*=7.6 Hz, H-1 of xyl), 5.45(1H, br s, H-12), 6.25 (1H, br s, H-1 of rha), ^13^C-NMR data.
